# Blood Biomarkers of Alzheimer's Disease and Gait Speed Trajectories in Community‐Dwelling Older Adults: A Cohort Study

**DOI:** 10.1002/jcsm.70365

**Published:** 2026-08-18

**Authors:** Javier Leal‐Martín, Giulia Grande, Caterina Gregorio, Amaia Calderón‐Larrañaga, Alice Margherita Ornago, Laura Fratiglioni, Martina Valletta, Claudia Fredolini, Anna‐Karin Welmer, Bengt Winblad, Davide Liborio Vetrano

**Affiliations:** ^1^ Aging Research Center, Department of Neurobiology, Care Sciences and Society Karolinska Institutet and Stockholm University Stockholm Sweden; ^2^ Division of Neurogeriatrics, Department of Neurobiology, Care Sciences and Society Karolinska Institutet Solna Sweden; ^3^ Stockholm Gerontology Research Center Stockholm Sweden; ^4^ Affinity Proteomics Stockholm, Science for Life Laboratory, Department of Protein Science School of Engineering Sciences in Chemistry, Biotechnology and Health (CBH), Royal Institute of Technology (KTH) Solna Sweden; ^5^ Division of Physiotherapy, Department of Neurobiology, Care Sciences and Society Karolinska Institutet Stockholm Sweden; ^6^ Women's Health and Allied Health Professionals Theme, Medical Unit Medical Psychology Karolinska University Hospital Stockholm Sweden; ^7^ Theme Inflammation and Aging Karolinska University Hospital Huddinge Sweden

**Keywords:** cognition, frailty, mobility limitations, neurodegeneration, physical performance, walking speed

## Abstract

**Background:**

Gait speed (GS) has been suggested as a predictor of incident dementia in old age. However, the mechanisms underlying this body–mind connection remain unclear, and it is still unknown whether trajectories of GS decline differ according to levels of blood biomarkers related to Alzheimer's disease (ad). This study aims to investigate the association between levels of seven blood biomarkers related to AD and long‐term GS changes in dementia‐free older adults living in the community.

**Methods:**

The present study included 1665 community‐dwelling adults ≥ 60 years, followed for 15 years, drawn from the Swedish National study on Aging and Care in Kungsholmen (SNAC‐K). GS (m·s^−1^) was assessed at baseline and at five subsequent follow‐up time points. Blood biomarkers, including serum amyloid‐*β*42 to amyloid‐*β*40 ratio (A*β*42/40), phosphorylated Tau181 (*p*‐Tau181) and Tau217 (*p*‐Tau217), total Tau (*t*‐Tau), neurofilament light chain (NfL) and glial fibrillary acidic protein (GFAP), were collected from peripheral venous blood samples at baseline. Linear mixed‐effects models were implemented to investigate the association between blood biomarkers and GS changes over time.

**Results:**

After adjusting for potential confounders, participants in the highest quartile of *p*‐Tau181 (Model I; 4th quartile *β*: −0.008, 95% CI: −0.013; −0.003), *p*‐Tau217 (Model I; 4th quartile *β*: −0.010, 95% CI: −0.015; −0.005), NfL (Model I; 4th quartile *β*: −0.013, 95% CI: −0.019; −0.006) and GFAP (Model I; 4th quartile *β*: −0.007, 95% CI: −0.013; −0.002) exhibited a steeper decline in GS over time, as compared with those in the lowest quartile. Notably, participants in these highest quartiles developed mobility limitations (GS < 0.8 m·s^−1^), on average, 2.3 to 6.0 years earlier. Of note, only the association between NfL and GS changes over time persisted after further adjusting for time‐varying global cognition.

**Conclusions:**

Higher blood biomarker levels of AD are associated with accelerated trajectories of GS decline and earlier onset of mobility limitations. Consequently, a faster decline in GS may reflect underlying neurodegeneration and indicate changes in brain health.

## 1 Introduction

Gait function is a key determinant of mobility that enables individuals to navigate and interact with their environment [1]. Among its features, gait speed (GS) stands out as a valid, reliable and sensitive clinical marker of overall health [2, 3, 4, 5] frequently used in the assessment of older adults' function.

The central nervous system plays a preeminent role in regulating GS [6]; thereby, optimal brain health is a prerequisite for its preservation. Ensuring adequate GS levels becomes important in later life, when maintaining mobility and physical independence is necessary for successful aging [7]. However, advancing age entails an increased risk of neurodegenerative conditions, such as Alzheimer's disease (ad), which challenge this goal [8]. In this regard, research in older adults confirms that an accelerated decline in GS is associated with cognitive impairment and incident dementia [6, 9, 10], and that such a decline may precede noticeable cognitive deterioration [11, 12, 13].

The biological mechanisms underlying this body–mind connection are not yet completely understood. Some literature has explored GS in relation to a panel of fluid‐based biomarkers associated with AD neuropathological changes [14, 15, 16, 17, 18]. However, research emphasis has been uneven across them, with no studies examining all these biomarkers within a single sample. While most studies have focused on amyloid‐*β* (A*β*) and neurofilament light chain (NfL) levels, biomarkers of neuroinflammation such as glial fibrillary acidic protein (GFAP) or Tau hyperphosphorylation such as phosphorylated Tau217 (*p*‐Tau217) [19], have been scarcely explored. Given that each ad biomarker reflects distinct pathogenic processes and stages, examining them in isolation has limited the ability to draw conclusions within a broader biological context [20]. Additionally, no study addressing this phenomenon has yet explored whether distinctive trajectories of GS decline can be identified in relation to increasing levels of AD‐related biomarkers.

Since blood‐based biomarkers have demonstrated strong predictive performance for ad and all‐case dementia in healthy individuals [17], exploring their association with GS trajectories may enhance our understanding of the mobility decline signature linked to neurodegeneration. It may also clarify the extent to which GS monitoring can serve as an indicator of brain health changes and how these changes might contribute to the earlier onset of mobility limitations. Therefore, the present study aims to investigate the relationship between levels of seven blood‐based AD‐related biomarkers and long‐term GS changes in a population‐based cohort of dementia‐free older adults living in the community.

## 2 Materials and Methods

### 2.1 Study Design and Sample

We utilized data from the Swedish National Study on Aging and Care in Kungsholmen (SNAC‐K), which includes a population‐based sample of community‐dwelling and institutionalized participants aged ≥ 60 years from the Kungsholmen neighbourhood in Stockholm, Sweden. This longitudinal, prospective cohort study performed its baseline assessment (wave 1) between 2001 and 2004, involving 11 different age cohorts (60, 66, 72, 78, 81, 84, 87, 90, 93, 96 and 99 + years). Follow‐up assessments have been conducted every 6 years for participants aged 60–78 years and every 3 years for those aged > 78 years. Of the participants initially invited to participate in SNAC‐K, 3363 (73%) enrolled at baseline.

**FIGURE 1:**
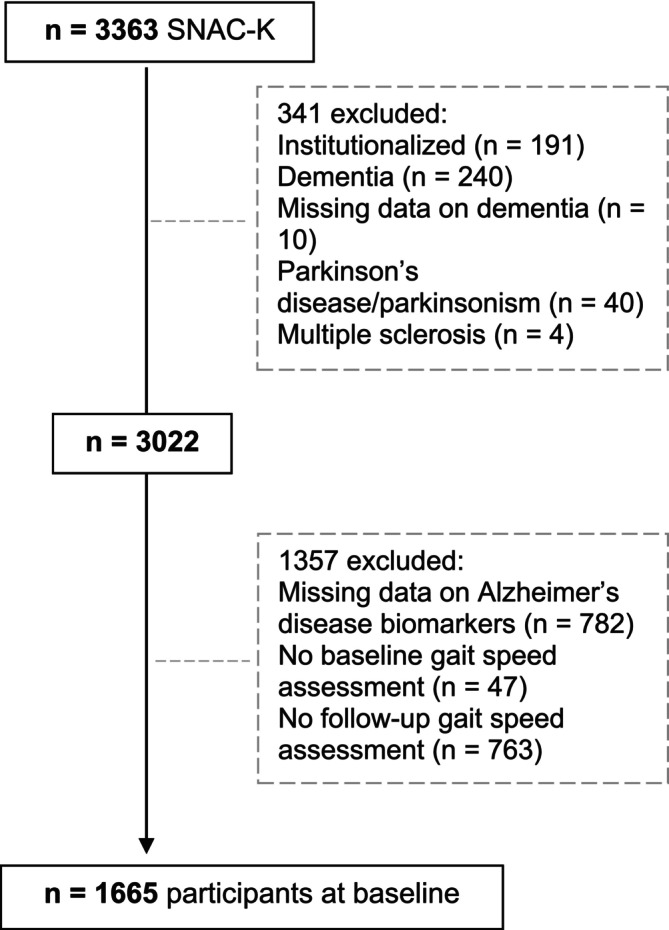
Flowchart of study participants.

This study included data from the first six waves of the SNAC‐K cohort, encompassing assessments up to the period between 2016 and 2019 (wave 6). From the total baseline sample, an eligible subset of 3022 participants was identified after excluding those who were institutionalized (*n* = 191), diagnosed with dementia (*n* = 240), Parkinson's disease (*n* = 40), multiple sclerosis (*n* = 4) as well as those participants with missing data on any of these variables (missing data on dementia, *n* = 10). From this eligible group, 1665 participants were included in the final analysis after further excluding participants with at least one missing AD blood biomarker value (*n* = 782), those lacking baseline GS data (*n* = 47) and those without GS data in at least one follow‐up (*n* = 763) (Figure 1). Those excluded were, on average, older, less educated and had more diseases (Table S1). The SNAC‐K protocols were approved by the Stockholm Regional Ethics Review Committee and the Swedish Ethical Review Authority and were conducted in compliance with the World Medical Association Declaration of Helsinki. Informed consent was obtained from all participants or their representatives in cases of cognitive impairment. The study findings are presented in accordance with the STROBE guidelines (Table S2).

### 2.2 Data Collection

Study participants underwent standardized evaluation procedures consisting of face‐to‐face interviews and clinical and laboratory assessments conducted by a multidisciplinary team of physicians, nurses and psychologists. Evaluations took place either at the Stockholm Gerontology Research Center or, for participants unable to attend the center, during home visits.

### 2.3 Gait Speed

Participants were asked to walk either 6 m or 2.4 m at their usual speed, with the distance determined based on their self‐assessed pace. Participants who rated themselves as fast or normal walkers completed the 6‐m walk, while those who rated themselves as slow or very slow walkers completed the 2.4‐m walk [21]. The 2.4‐m walk was also used if the assessment was conducted in a restricted space, such as participants' homes or nursing homes. The timed walking distance (6 m or 2.4 m) was preceded by a 1 m acceleration phase and followed by a 1 m deceleration phase. This test was supervised by trained nurses, who recorded the time (s) required for participants to complete the assigned distance (m). GS was then calculated in meters per second (m·s^−1^) and categorized, only at baseline, as either slow GS (< 0.8 m·s^−1^) or fast GS (≥ 0.8 m·s^−1^). A 0.8 m·s^−1^ cutoff was used as GS below this value has shown to predict survival below the median in community‐dwelling older adults [3], and is frequently used as a proxy of mobility limitations [22]. GS was evaluated at both baseline and follow‐ups.

### 2.4 Blood‐Based Biomarkers and Apolipoprotein E (APOE) ε4 Genotype

Peripheral venous blood samples were collected at baseline (fasting was not compulsory) and upon centrifugation, serum aliquots were stored at the Karolinska Institutet BioBank at −80°C in cryogenic storage vials until analysis. For blood biomarkers, protein quantification was conducted at the Affinity Proteomics Stockholm Unit (SciLifeLab), using the Quanterix SR‐X (software version 1.2.0) Biomarker Detection System. Serum NfL and GFAP were measured using Simoa Neuro 2‐plex B Kit. Serum amyloid‐*β*40 (A*β*40), amyloid‐*β*42 (A*β*42) and total Tau (*t*‐Tau) were measured using Simoa Neuro 3‐plex A Kit. Serum phosphorylated Tau181 (*p*‐Tau181) was measured using Simoa *p*‐Tau181 Advantage V2 Kit, and *p*‐Tau217 using Simoa ALZpath *p*‐Tau217 Advantage PLUS. For each kit, 25 μL of sample were diluted 1:4 and the assays were performed according to manufacturer instructions or internal validation protocols. The Quanterix instrument provides average enzyme per bead (AEB) values for calibrators, controls and samples across all proteins. Curve fitting, concentration extrapolation and graphical representation are automatically performed within the Quanterix SR‐X software using the calibrators (known concentration series of an analyte) and a four‐parameter logistic (4PL) curve fit. Precision was estimated for each assay. The within‐run coefficient of variation was calculated using a triplicate serum pool (from SNAC‐K samples), together with control 1 and control 2 from the Quanterix kits included in each run. The between‐run coefficient of variation was determined on the basis of concentration values for the serum pool, control 1 and control 2 across all runs. Details of the within‐run and between‐run coefficient of variation can be found in Table S3. Data below the limit of detection were replaced, using a not missing at random strategy and single‐value imputation, with a value of 0 (imputed measurements: *n* = 6 for A*β*42, *n* = 13 for *p*‐Tau181, *n* = 3 for *p*‐Tau217 and *n* = 12 for *t*‐Tau). The A*β*42 to A*β*40 ratio (A*β*42/40) was then calculated. Finally *APOE* genotyping was performed on DNA isolated from peripheral blood samples. Participants carrying at least one *APOE* ε4 allele were categorized as ε4 carriers, whereas those lacking the allele were identified as non‐carriers. Both AD blood‐based biomarkers and *APOE* ε4 genotype were obtained at baseline.

### 2.5 Covariates

Baseline sociodemographic, anthropometric, functional, lifestyle and clinical data were collected. Sociodemographic and anthropometric variables included sex, age, civil status, educational attainment and body mass index (BMI). Civil status was determined as either having a partner or not. Education was categorized as elementary school, high school or university level and above. Functional independence was assessed as the ability to carry out activities of daily living (ADLs), calculated as the sum of impaired domains across six basic self‐care tasks: bathing, dressing, toileting, continence, transferring from bed and eating. Physical activity levels were assessed through a questionnaire on the frequency and intensity of weekly exercise. Participants were classified into two groups: (1) inadequate, defined as 2–3 times or less per month of light and/or moderate‐to‐vigorous exercise and (2) health‐ or fitness‐enhancing, defined as engaging in either light exercise several times per week or moderate‐to‐vigorous exercise several times per week. Chronic conditions were identified via clinical interviews, physical examinations, laboratory assessments, medication records and linkage to the National Patient Register using the diagnostic codes from the International Classification of Diseases, 10th revision (ICD‐10). Among these conditions, chronic kidney disease, hypertension, diabetes, cerebrovascular disease, heart failure, atrial fibrillation, ischemic heart disease, cerebrovascular disease were selected as covariates. The number of medications was also recorded during examinations and through self‐reported and nurse‐administered questionnaires.

Dementia and global cognition were assessed at both baseline and follow‐ups. Dementia was diagnosed according to the Diagnostic and Statistical Manual of Mental Disorders, 4th Edition (DSM‐IV), using a previously established three‐step protocol [23]. Incident dementia was defined as a new diagnosis during the follow‐up period. Global cognitive function was assessed using the Mini‐Mental State Examination (MMSE), which ranges from 0 to 30 points. The MMSE consists of two sections [24]: the first evaluates orientation, memory and attention; the second assesses naming, the ability to follow verbal and written commands, spontaneous sentence writing and copying a complex polygon resembling the Bender‐Gestalt figure.

### 2.6 Statistics

Baseline characteristics of participants were reported using number with percentage (%), mean with standard deviations (SD) or median with interquartile ranges (IQR). To compare the characteristics between included/excluded participants and by GS in the included sample, Chi‐square test for categorical variables and either Student's *t*‐test or Mann–Whitney U test for continuous variables were performed. For standardized comparisons, biomarker values were z‐scored.

To investigate the relationship between baseline serum AD biomarker levels and changes in GS over time, linear mixed‐effects models were employed. These models incorporated random intercepts, random slopes and interaction terms between both biomarkers and covariates and follow‐up time. AD biomarker values were categorized into quartiles to allow characterization of GS trajectories across increasing biomarker levels, while minimizing the influence of skewed biomarker distributions and extreme values. This biomarker operationalization is also consistent with previous studies based on the SNAC‐K cohort investigating dementia prediction [17]. Three regression models were conducted, with progressively refined covariate adjustments. Model I accounted for baseline age, sex, education, BMI, physical activity status, number of medications, chronic kidney disease, hypertension, diabetes, cerebrovascular disease, heart failure, atrial fibrillation, ischemic heart disease and other cardiovascular diseases. Model II extended the adjustments by including *APOE* ε4 genotype status. Further analyses (Model III) included time‐varying MMSE. Model III is proposed to mainly test the hypothesis that cognitive function might partly mediate the association between AD pathology and GS decline. The 4th quartile was set as a reference for A*β*42/40, while the 1st quartile was set as a reference for *t*‐Tau, *p*‐Tau181, *p*‐Tau217, NfL and GFAP. For biomarkers that were significantly associated with GS decline in Model II, additional exploratory analyses were conducted to estimate the mean follow‐up time (95% CI) at which participants in the most disadvantaged quartile reached a GS < 0.8 m·s^−1^, used as a proxy for the onset of mobility limitations. These estimates were derived from the GS predicted trajectories obtained from Model II. Bonferroni‐adjusted *p*‐values and CI were additionally derived for the biomarker‐by‐follow‐up time interaction terms to account for multiple quartile‐based comparisons in each biomarker‐specific model.

Finally, sensitivity analyses were performed to discard reverse causality and evaluate the impact of additional exclusion criteria. Specifically, we excluded participants with: (1) slow GS at baseline (*n* = 268), (2) cerebrovascular disease at baseline (*n* = 74), (3) imputed biomarkers (*n* = 6 for A*β*42 [A*β*42/40], *n* = 13 for *p*‐Tau181, *n* = 3 for *p*‐Tau217 and *n* = 12 for *t*‐Tau) and (4) incident dementia (*n* = 266). As in the primary models, these analyses incorporated random intercepts, random slopes and interaction terms between both biomarkers and covariates and follow‐up time. Covariate adjustments were applied as specified in Model I, Model II and Model III.

All statistical analyses were conducted using R software (4.4.2), with statistical significance defined as *p* < 0.05.

## 3 Results

### 3.1 Sample Characteristics

**FIGURE 2:**
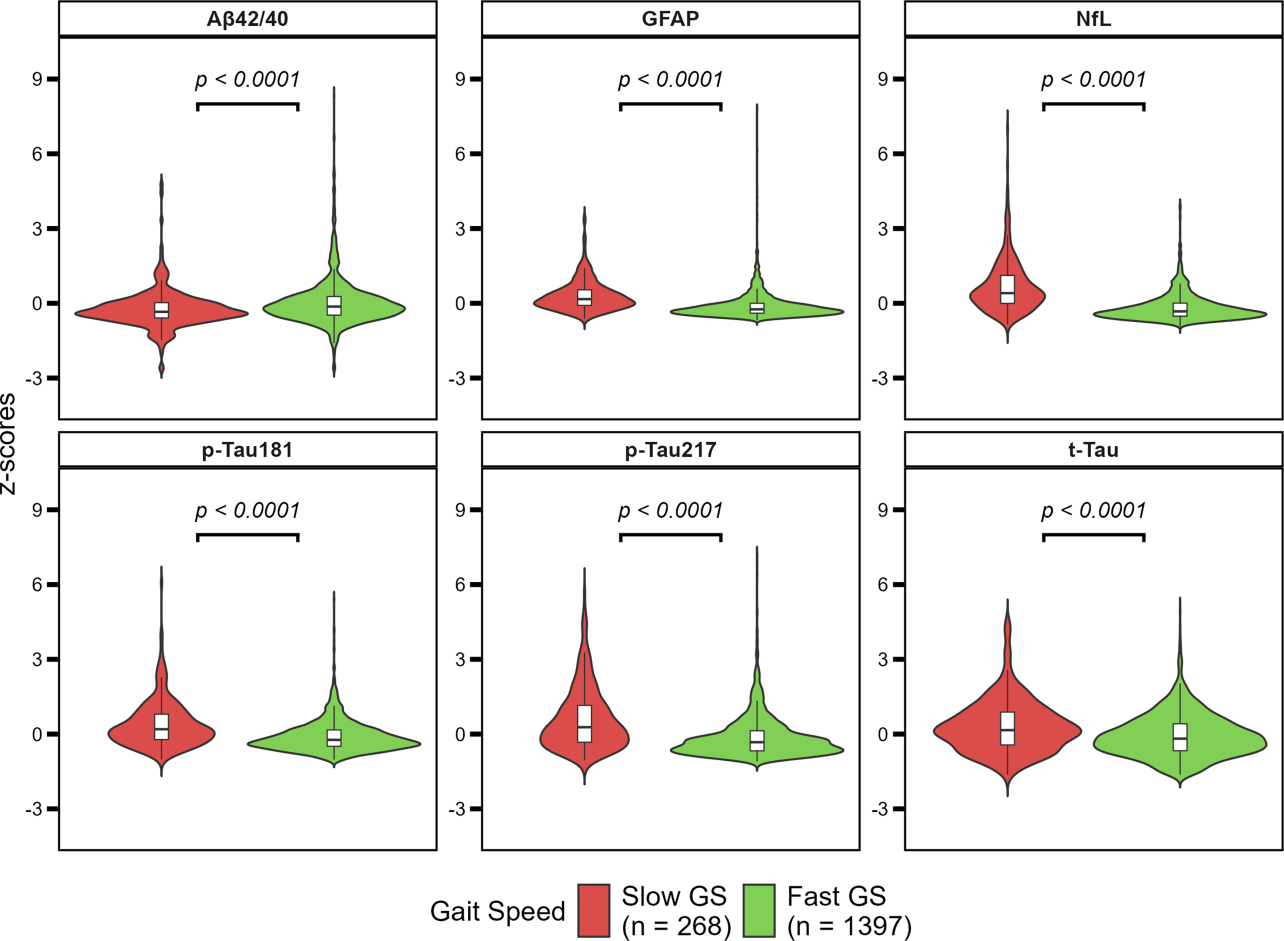
Blood biomarkers differences between fast and slow gait speed groups. A*β*42/40 = amyloid‐*β*42 to amyloid‐*β*40 ratio; GFAP = glial fibrillary acidic protein; NfL = neurofilament light chain; *p*‐Tau181 = phosphorylated Tau181; *p*‐Tau217 = phosphorylated Tau217; *t*‐Tau = total Tau.

The characteristics of the included sample are displayed in Table 1. The 1665 participants had a mean age of 71.05 ± 9.87 years, 62.52% were female, and a median follow‐up time of 11.41 (IQR: 5.95, 12.02). The mean MMSE score was 28.92 ± 1.48, with 38.92% reporting a university‐level education or above. The mean GS was 1.12 ± 0.38 m·s^−1^, and 29.13% of participants were *APOE* ε4 carriers. Compared to participants with slow GS (*n* = 268), those with a faster GS (*n* = 1397) were significantly younger, more physically active, and exhibited fewer diseases and greater ability to perform ADLs. They were also more highly educated, demonstrated better cognitive performance, and had significantly higher A*β*42/40, along with lower values of *p*‐Tau181, *p*‐Tau217, *t*‐Tau, NfL and GFAP (Figure 2). Fast GS participants also included a significantly higher proportion of *APOE* ε4 carriers.

**TABLE 1:** Baseline characteristics of the included sample, stratified by gait speed.

	Total (*n* = 1665)	Slow GS (*n* = 268)	Fast GS (*n* = 1397)	*p*
*Sociodemographic characteristics*				
Age (years), mean (SD)	71.05 (9.87)	82.00 (8.71)	68.95 (8.62)	**< 0.0001**
BMI (kg·m^−2^), mean (SD)	25.90 (3.98)	25.59 (5.55)	25.95 (3.64)	**0.021**
Female, *n* (%)	1041 (62.52)	212 (79.10)	829 (59.34)	**< 0.0001**
Education				**< 0.0001**
Elementary school, *n* (%)	210 (12.61)	67 (25.00)	143 (10.24)	
High school, *n* (%)	807 (48.47)	147 (54.85)	660 (47.24)	
University or above, *n* (%)	648 (38.92)	54 (20.15)	594 (42.52)	
Civil status				**< 0.0001**
Partnered, *n* (%)	859 (51.59)	66 (24.63)	793 (56.76)	
Unpartnered, *n* (%)	805 (48.35)	202 (75.37)	603 (43.16)	
*Physical activity level*				**< 0.0001**
Inadequate, *n* (%)	376 (22.58)	141 (52.61)	235 (16.82)	
Health or fitness‐enhancing, *n* (%)	1289 (77.42)	127 (47.39)	1162 (83.18)	
*Cognitive status*				
MMSE score, mean (SD)	28.92 (1.48)	27.87 (1.90)	29.12 (1.29)	**< 0.0001**
*Clinical assessments*				
Number of chronic diseases, mean (SD)	3.51 (2.22)	5.50 (2.49)	3.13 (1.94)	**< 0.0001**
Number of medications, mean (SD)	3.37 (3.11)	5.59 (3.88)	2.94 (2.75)	**< 0.0001**
Depression and mood disorders, *n* (%)	129 (7.75)	32 (11.94)	97 (6.94)	**0.0074**
Heart failure, *n* (%)	95 (5.71)	49 (18.28)	46 (3.29)	**< 0.0001**
Ischemic heart disease, *n* (%)	190 (11.41)	60 (22.39)	130 (9.31)	**< 0.0001**
Atrial fibrillation, *n* (%)	103 (6.19)	39 (14.55)	64 (4.58)	**< 0.0001**
Other cardiovascular disease, *n* (%)	31 (1.86)	11 (4.10)	20 (1.43)	**0.0066**
Hypertension, *n* (%)	1138 (68.35)	199 (74.25)	939 (67.22)	**0.028**
Cerebrovascular disease, *n* (%)	74 (4.44)	28 (10.45)	46 (3.29)	**< 0.0001**
Chronic kidney disease, *n* (%)	479 (28.77)	155 (57.84)	324 (23.19)	**< 0.0001**
Diabetes, *n* (%)	124 (7.45)	33 (12.31)	91 (6.51)	**0.0015**
Obesity, *n* (%)	230 (13.81)	41 (15.30)	189 (13.53)	0.50
Asthma, COPD, emphysema, bronchitis, *n* (%)	148 (8.89)	39 (14.55)	109 (7.80)	**0.0006**
Dorsopathies, *n* (%)	113 (6.79)	34 (12.69)	79 (5.65)	**< 0.0001**
Inflammatory arthropathies, *n* (%)	54 (3.24)	21 (7.84)	33 (2.36)	**< 0.0001**
Osteoarthritis degenerative joint disease, *n* (%)	211 (12.67)	56 (20.90)	155 (11.10)	**< 0.0001**
Other musculoskeletal joint disease, *n* (%)	90 (5.41)	29 (10.82)	61 (4.37)	**< 0.0001**
*APOE* ε4 carrier status, *n* (%)	485 (29.13)	61 (22.76)	424 (30.35)	**0.010**
*Physical function*				
Gait speed (m·s^−1^), mean (SD)	1.12 (0.38)	0.45 (0.21)	1.25 (0.25)	**< 0.0001**
ADLs				**< 0.0001**
0 difficulties, *n* (%)	1632 (98.02)	246 (91.79)	1386 (99.21)	
≥ 1 difficulties, *n* (%)	33 (1.98)	22 (8.21)	11 (0.79)	

*Note:* All variables are presented as Mean (SD) or *n* (%). *p* values depict a comparison between participants with a slow gait speed (< 0.8 m·s^−1^) and a fast gait speed (≥ 0.8 m·s^−1^), with bold values indicating *p* < 0.05. All the statistical tests were two‐sided. A mismatch between total numbers and sample sizes is due to missing data. Missing values for participants with slow gait speed: BMI = 26; *APOE* ε4 = 4. Missing values for participants with fast gait speed: BMI = 4; civil status = 1; MMSE = 2; A*POE* ε4 = 7.

Abbreviations: ADLs = activities of daily living; *APOE* ε4 = apolipoprotein E isoform 4; BMI = body mass index; GS = gait speed; MMSE = mini‐mental state examination; SD = standard deviation.

### 3.2 Blood Biomarkers Levels and GS Trajectories

Compared to the participants in the lowest reference quartiles (Table 2, Model I), a faster decline in GS was observed in those in the highest quartile of *p*‐Tau217 (4th quartile *β*: −0.010; 95% CI: −0.015, −0.005) and GFAP (4th quartile *β*: −0.007; 95% CI: −0.013, −0.002), as well as those in the two highest quartiles of *p*‐Tau181 (3rd quartile *β*: −0.005; 95% CI: −0.009, −0.0004; 4th quartile *β*: −0.008; 95% CI: −0.013, −0.003) and NfL (3rd quartile, *β*: −0.008; 95% CI: −0.013, −0.003; 4th quartile, *β*: −0.013; 95% CI: −0.019, −0.006).

**TABLE 2:** Association between quartile‐based ad‐related biomarkers and annual change in gait speed (m·s^−1^) over the 15‐year follow‐up.

	Model I			Model II			Model III		
	*β*	(95% CI)	*p*	*β*	(95% CI)	*p*	*β*	(95% CI)	*p*
A*β*42/40									
1st quartile	−0.002	(−0.007; 0.003)	0.39	−0.002	(−0.006; 0.003)	0.45	−0.001	(−0.005; 0.004)	0.74
2nd quartile	0.001	(−0.003; 0.006)	0.56	0.002	(−0.003; 0.006)	0.43	0.003	(−0.002; 0.007)	0.22
3rd quartile	0.003	(−0.001; 0.008)	0.13	0.003	(−0.001; 0.008)	0.14	0.003	(−0.001; 0.008)	0.11
^a^4th quartile	—	—	—	—	—	—	—	—	—
*p*‐Tau181									
^a^1st quartile	—	—	—	—	—	—	—	—	—
2nd quartile	0.000	(−0.005; 0.004)	0.82	0.000	(−0.005; 0.004)	0.89	0.000	(−0.004; 0.004)	0.99
3rd quartile	**−0.005**	**(−0.009; −0.0004)**	**0.034**	−0.004	(−0.009; 0.000)	0.054	−0.004	(−0.008; 0.001)	0.095
4th quartile	**−0.008**	**(−0.013; −0.003)**	**0.0028**	**−0.007**	**(−0.013; −0.002)**	**0.0057**	−0.004	(−0.009; 0.001)	0.13
*p*‐Tau217									
^a^1st quartile	—	—	—	—	—	—	—	—	—
2nd quartile	−0.002	(−0.007; 0.002)	0.32	−0.002	(−0.007; 0.002)	0.32	−0.002	(−0.006; 0.003)	0.44
3rd quartile	−0.002	(−0.006; 0.003)	0.48	−0.001	(−0.006; 0.003)	0.57	−0.001	(−0.005; 0.003)	0.72
4th quartile	**−0.010**	**(−0.015; −0.005)**	**0.0002**	**−0.009**	**(−0.015; −0.004)**	**0.0008**	−0.005	(−0.010; 0.000)	0.055
*t*‐Tau									
^a^1st quartile	—	—	—	—	—	—	—	—	—
2nd quartile	0.000	(−0.004; 0.005)	0.89	0.000	(−0.004; 0.005)	0.84	0.000	(−0.004; 0.004)	0.94
3rd quartile	−0.001	(−0.006; 0.003)	0.55	−0.001	(−0.006; 0.003)	0.54	−0.001	(−0.006; 0.003)	0.56
4th quartile	0.000	(−0.005; 0.005)	0.98	0.000	(−0.005; 0.005)	0.98	0.000	(−0.004; 0.005)	0.92
NfL									
^a^1st quartile	—	—	—	—	—	—	—	—	—
2nd quartile	−0.003	(−0.008; 0.001)	0.14	−0.003	(−0.007; 0.001)	0.16	−0.003	(−0.007; 0.001)	0.12
3rd quartile	**−0.008**	**(−0.013; −0.003)**	**0.0013**	**−0.008**	**(−0.013; −0.003)**	**0.0014**	**−0.007**	**(−0.012; −0.002)**	**0.0030**
4th quartile	**−0.013**	**(−0.019; −0.006)**	**0.0001**	**−0.012**	**(−0.019; −0.006)**	**0.0002**	**−0.008**	**(−0.014; −0.002)**	**0.014**
GFAP									
^a^1st quartile	—	—	—	—	—	—	—	—	—
2nd quartile	0.001	(−0.003; 0.005)	0.64	0.001	(−0.003; 0.005)	0.65	0.001	(−0.003; 0.005)	0.72
3rd quartile	−0.002	(−0.007; 0.003)	0.37	−0.002	(−0.007; 0.003)	0.38	−0.001	(−0.006; 0.003)	0.54
4th quartile	**−0.007**	**(−0.013; −0.002)**	**0.011**	**−0.007**	**(−0.013; −0.002)**	**0.013**	−0.003	(−0.009; 0.002)	0.23

*Note:* This table depicts the results from linear mixed‐effect models with longitudinal gait speed (m·s^−1^) as the outcome and quartile‐based AD‐related biomarkers. Model I: model including age, sex, education, BMI, physical activity, number of drugs, chronic kidney disease, diabetes, hypertension, cerebrovascular disease, heart failure, atrial fibrillation, ischemic heart disease and other cardiovascular diseases. Model II: model I + *APOE* ε4. Model III: model II + time‐varying Mini‐Mental State Examination scale score. Bold indicates *p* < 0.05.

Abbreviations: A*β*42/40 = amyloid‐*β*42 to amyloid‐*β*40 ratio; CI = confidence interval; GFAP = glial fibrillary acidic protein; NfL = neurofilament light chain; *p*‐Tau181 = phosphorylated Tau181; *p*‐Tau217 = phosphorylated Tau217; *t*‐Tau = total‐Tau.

^a^ Reference quartile.

**FIGURE 3:**
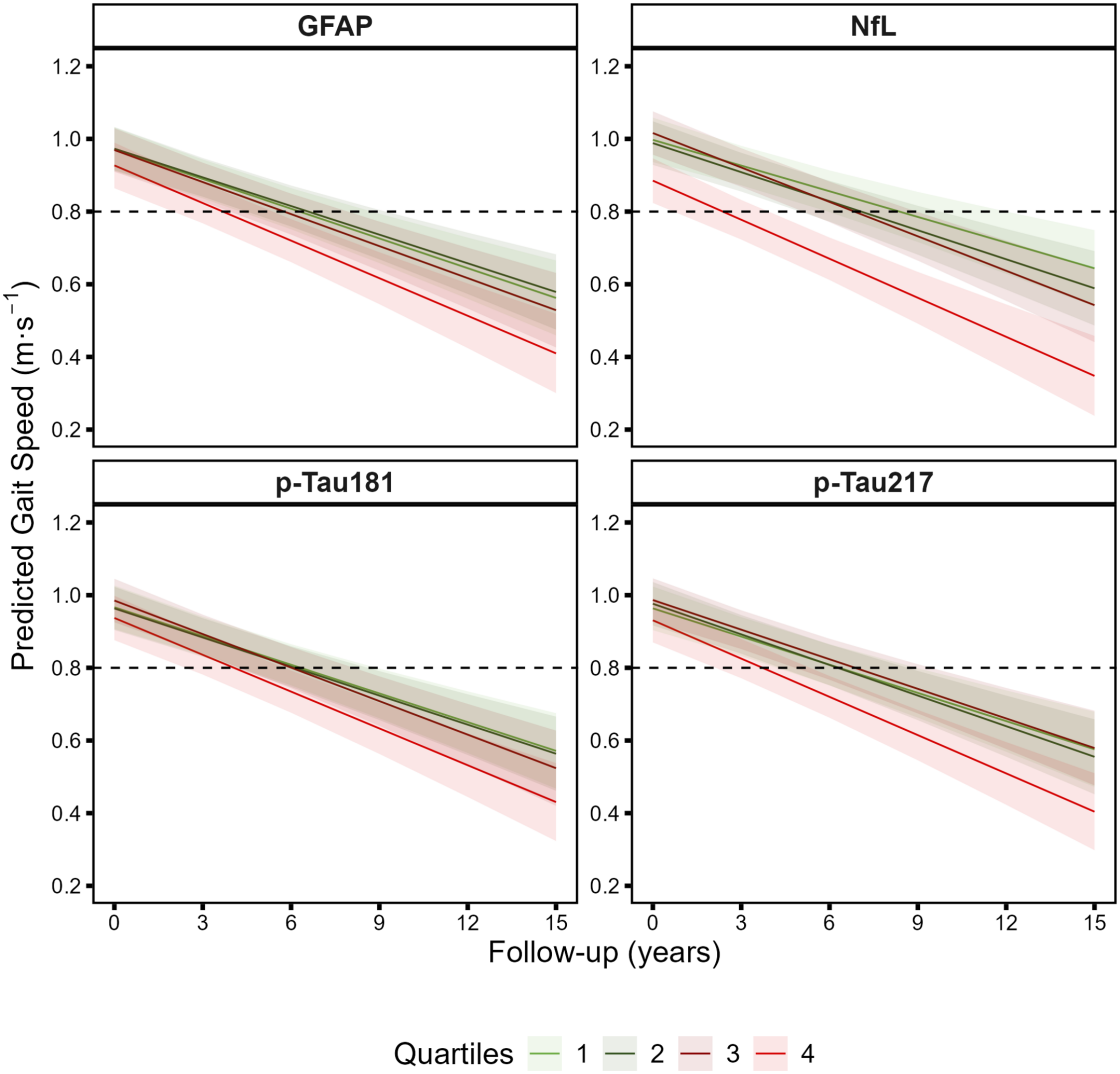
Longitudinal trajectories of gait speed by quartiles of AD‐related blood biomarkers. Predicted gait speed decline from linear mixed models is adjusted for age, sex, education, body mass index, physical activity, number of medications, chronic kidney disease, hypertension, diabetes, cerebrovascular disease, heart failure, atrial fibrillation, ischemic heart disease, other cardiovascular disease and *APOE* ε4 (Model II). GFAP = glial fibrillary acidic protein; NfL = neurofilament light chain; *p*‐Tau181 = phosphorylated Tau 181; *p*‐Tau217 = phosphorylated Tau217. The horizontal dashed line indicates a 0.8 m·s^−1^ threshold for mobility limitations.

After adjustment for *APOE* ε4 genotype (Table 2, Model II), the association for *p*‐Tau181 weakened, with only participants whose biomarker levels were in the 4th quartile remaining significantly associated with GS changes. As a result, participants in the highest biomarker quartile of *p*‐Tau181, *p*‐Tau217, NfL and GFAP developed mobility limitations (GS < 0.8 m·s^−1^), on average, after 4.06 years (95% CI: 2.07, 6.46), 3.72 years (95% CI: 1.84, 5.96), 2.38 years (95% CI: 0.61, 4.49) and 3.68 years (95% CI: 1.71, 6.04) of follow‐up, respectively. This is approximately 2–6 years earlier than participants in the lowest quartiles (Figure 3).

Further accounting for time‐varying MMSE (Table 2, Model III) led to additional attenuation, and the associations for *p*‐Tau181, *p*‐Tau217 and GFAP weakened and were no longer significant. Only participants in the two highest quartiles of NfL continued exhibiting a significantly faster GS decline compared to those in the 1st quartile, albeit with an attenuation (Table 2, Model III).

Consistent with these findings, Bonferroni‐adjusted models showed similar patterns of associations except for Model I *p*‐Tau181, which no longer reached statistical significance (Table S4).

Finally, sensitivity analyses yielded similar results after excluding participants with slow GS, cerebrovascular disease at baseline and imputed ad biomarkers (Tables S5–S7). However, associations for *p*‐Tau181, *p*‐Tau217, GFAP and the highest NfL quartile became nonsignificant after excluding participants with incident dementia, regardless of adjustments (Table S8).

## 4 Discussion

The present study attempts to comprehensively investigate the association between levels of blood biomarkers related to AD with long‐term GS trajectories in community‐dwelling older adults free from dementia. After adjusting for relevant confounders, including *APOE* ε4 genotype, participants with the highest serum levels of *p*‐Tau181, *p*‐Tau217, NfL and GFAP exhibited a steeper trajectory of GS decline. As a result, these participants experienced mobility limitations (GS < 0.8 m·s^−1^), on average, 2 to 6 years earlier than those with the lowest biomarker levels. However, after controlling for time‐varying cognitive status, only the two highest quartiles of serum NfL remained significantly associated with GS decline, compared to the lowest quartile. These findings suggest that cognitive function may drive, to some extent, the relationship between AD pathology and GS. However, a higher burden of axonal damage, as reflected by serum NfL and arguably of vascular nature or reflecting other neurodegenerative processes, may relate more strongly than other pathological processes to an accelerated trajectory of GS decline.

Compared to previous studies investigating blood biomarkers related to AD and GS, our results suggest a more complex and nonlinear relationship. Before adjusting for *APOE* ε4 genotype, *p*‐Tau181 related to GS across a broader range (3rd and 4th quartiles) than *p*‐Tau217 (4th quartile only). However, after adjustment for *APOE* ε4 genotype, both *p*‐Tau181 and *p*‐Tau217 exhibited similar patterns of association. Focusing on *p*‐Tau217, our study provides novel evidence on its relationship with gait function, complementing earlier investigations in this cohort by Ornago et al. [25], who found that higher levels of this biomarker were significantly associated with faster decline in both upper and lower limb muscle strength. Among studies exploring *p*‐Tau181, only the one by Ali et al. [26] reported a similar relationship with GS, although using continuous biomarker measures. Nevertheless, direct comparison with other previous studies is limited due to methodological differences. Most of them followed a cross‐sectional design, and all except that of Ali et al. [26] assessed GS using the Timed Up and Go test or its variants, which include additional components such as sit‐to‐stand transitions, seating posture and changes in direction [27, 28, 29].

Similarly, participants in the highest quartile of GFAP levels exhibited a significantly faster decline in GS, suggesting that reactive astrogliosis and neuroinflammation may also be linked to mobility function [30, 31]. To date, only two studies have investigated the relationship between GFAP and GS, yielding contradictory results. Specifically, the longitudinal study by Nadkarni et al. [32] found no association after adjusting for potential confounders. In contrast, the other study, although cross‐sectional, reported an association that persisted after controlling for age, sex and other covariates [26].

Furthermore, NfL emerged as the biomarker most strongly associated with GS changes, demonstrating a significant inverse relationship even at more moderate levels (3rd quartile), which remained after adjusting for *APOE* ε4 status. These findings support axonal degeneration as a more comprehensive indicator of mobility deterioration. Notably, participants in the highest NfL quartile were more likely to experience earlier mobility limitations, defined as having a GS < 0.8 m·s^−1^, occurring, on average, 1.4 years earlier than those in the highest quartiles of *p*‐Tau181, *p*‐Tau217 and GFAP, and 6.0 years earlier than those in the lowest NfL quartile. This is consistent with prior investigations of NfL and GS, such as the study by Ali et al. [26], which reported that NfL was more strongly associated with GS than either p‐Tau181 or GFAP levels. Similarly, the longitudinal study by Nadkarni et al. [32] reported an association between higher serum NfL levels and greater annual decline in GS, even after adjusting for sex, age, education, BMI and other clinical parameters.

Since cognitive status may mediate the association between AD‐related biomarkers and GS, we repeated the regression models while controlling for time‐varying global cognition (MMSE). Notably, the associations for *p*‐Tau181, *p*‐Tau217 and GFAP weakened and became non‐significant, whereas the associations for the two highest quartiles of NfL remained. A similar pattern was observed when participants with incident dementia were excluded, even without adjusting for global cognition. Elevated levels of *p*‐Tau and GFAP depict amyloid pathology and glial activation, and can be indicative of either a high risk of AD‐related cognitive impairment or help substantiate presumed prodromal dementia [15]. Therefore, cognition could be acting as a potential mediator in the relationship between more AD‐specific biomarkers and GS, particularly among participants developing dementia. Still, some studies such as Ali et al. [26] have reported associations between continuous levels of *p*‐Tau181 and GFAP with GS, regardless of cognitive status. In contrast, NfL, considered a broader biomarker of neurodegeneration, may also be capturing the effects on GS of inconspicuous disorders or injuries, whether related to AD or not. The underlying cause, which may be vascular, metabolic or otherwise, could originate in either the central or peripheral nervous system, yet may not exclusively affect neural structures involved in the cognitive component of gait [6, 33, 34]. Importantly, GS decline unrelated to cognition may still signal an increased risk of future cognitive deterioration or dementia. Supporting this, Collyer et al. [35] showed that, although individuals with cognition decline only and dual decliners exhibited the highest cumulative incidence of dementia, those classified as gait decliners only still showed an increased incidence of dementia compared to non‐decliners, and even surpassed cognition decliners only when operationalized through processing speed or verbal fluency. Similarly, Skillbäck et al. [11] identified a faster slowing of GS in individuals who subsequently experienced cognitive decline, up to 9 years before this cognitive decline was detectable, compared to those remaining cognitively normal. Therefore, accelerated GS decline in cognitively intact older adults may also reflect underlying neurodegeneration and an increased risk of future cognitive decline and dementia.

Lastly, among all the biomarkers explored in our study, levels of A*β*42/40 and *t*‐Tau were not associated with long‐term GS trajectories regardless of covariate adjustments. These findings align with the majority of prior evidence reported in the literature [26, 32, 36, 37, 38, 39]. Multiple factors may explain this lack of association, including the low concentrations of these biomarkers in blood, weaker blood‐to‐brain correlations, limited assay sensitivity and greater susceptibility to degradation or peripheral metabolism [16, 40].

Several limitations of this study should be acknowledged. First, as an observational study, causal relationships cannot be inferred from the analyses. Furthermore, GS was not adjusted for leg length, as this measurement was unavailable. Additionally, the use of two walking distances (2.4 m and 6 m) may have introduced some degree of measurement heterogeneity. However, previous studies have reported highly comparable GS estimates across short walking distances, including 2.4 m and 6 m [21, 41]. Moreover, evidence suggests that differences between GS assessments may be more strongly influenced by protocol characteristics, such as static versus dynamic start and stop conditions [41]. In the present study, these conditions were kept consistent across distances (i.e., 1‐m acceleration and deceleration phases). In addition, reliance on serum‐based biomarkers may introduce variability compared to plasma, cerebrospinal fluid or imaging markers [18]. Moreover, participants excluded from the eligible sample were generally older and had a greater disease burden, which may have led to an underestimation of the observed associations and may have limited their generalizability to frailer or more clinically vulnerable older adults. In addition, statistical testing relied on multiple quartile‐based contrasts within each biomarker model. Although residual type I error cannot be entirely excluded, Bonferroni‐adjusted *p*‐values and CIs yielded results mostly consistent with the primary models. Lastly, the SNAC‐K participants were recruited from a high‐income and highly educated neighbourhood, which should be considered when generalizing the findings to other populations or regions.

On the other hand, this study has several strengths. Its population‐based design and large sample size strengthen the robustness and public health relevance of the findings. The 15‐year follow‐up period allows for the observation of dynamic GS changes and the identification of trends that cross‐sectional studies might overlook. In fact, to the best of our knowledge, no other study addressing this objective has followed participants over such an extended period. Furthermore, the inclusion of a well‐characterized cohort, extensively assessed in terms of clinical and demographic information, enables the adjustment for multiple relevant confounding factors, thereby strengthening the reliability of the findings. Most significantly, the study makes a novel contribution to the literature by investigating, within a single aging cohort, the broadest range of biomarkers commonly related to AD and neurodegeneration in relation to GS. It further delineates distinctive trajectories of GS decline based on quartile‐defined biomarker categories and, uniquely, evaluates *p*‐Tau217 within this framework, providing novel perspectives on the mechanisms driving mobility deterioration and opening new avenues for advancing our understanding of AD pathophysiology.

In conclusion, the present study found that community‐dwelling older adults free from dementia with high levels of *p*‐Tau181, *p*‐Tau217, NfL and GFAP exhibit faster decline in GS over a 15‐year follow‐up. Moreover, this association does not appear to be affected by cognitive status when related to general axonal degeneration, as indicated by the robust associations with NfL. These findings suggest that accelerated GS decline may reflect underlying neurodegeneration or ad‐specific pathology and support GS as a potential indicator of brain health in old age. Nevertheless, further research is needed to clarify the clinical significance of these findings.

## Funding

Data collection of the Swedish National study on Aging and Care (SNAC‐K) was supported by the Swedish Research Council (ongoing/current grant: 2021‐00178); the Swedish Ministry of Health and Social Affairs; the participating County Councils and Municipalities. DLV was supported by the Swedish Research Council (project number 2021‐03324), the Karolinska Institutet Strategic Research Area in Epidemiology and Biostatistics in 2021 and 2023 and the Karolinska Institutet Strategic Research Area Neuroscience in 2025 and the Karolinska Institutet Committee for Research (consolidator grant 2026). BW was supported by the Margaretha af Ugglas' foundation. CG was supported by the Loo och Hans Ostermans Stiftelse för medicinsk forskning. ACL receives funding from the Swedish Research Council (project number 2021‐06398), the Swedish Research Council for Health, Working Life and Welfare (project numbers 2024‐01830 and 2021‐00256), Karolinska Institutet's Strategic Research Area in Epidemiology and Biostatistics SFOepi (consolidator bridging grant, 2023) and Alzheimerfonden (AF‐1010573, 2024).

## Ethics Statement

The SNAC‐K protocols were approved by the Stockholm Regional Ethics Review Committee (ethical permit approval numbers KI 01‐114, 2001‐114, 2004‐929/3, 2007/279‐31, 2010/2:4, 2013/3:6, 2016/730‐31/1), and were conducted in compliance with the World Medical Association Declaration of Helsinki.

## Consent

All subjects provided informed consent.

## Conflicts of Interest

The authors declare no conflicts of interest.

## Supporting information


**Table S1:** Eligible sample, excluded sample, and included sample.
**T** **able S2:** STROBE Statement—Checklist of items that should be included in reports of cohort studies.
**Table S3:** Intra‐assay and inter‐assay precision expressed as the coefficient of variation (%).
**Table S4:** Associations between quartile‐based AD‐related biomarkers and annual change in gait speed (m·s^−1^) over the 15‐year follow‐up, with Bonferroni‐adjusted *p*‐values and CI for biomarker‐by‐follow‐up time interaction terms.
**T** **able S5:** Associations between quartile‐based AD‐related biomarkers and annual change in gait speed (m·s^−1^) over the 15‐year follow‐up, excluding those participants with GS < 0.8 m·s^−1^ at baseline (*n* = 268).
**T** **able S6:** Associations between quartile‐based AD‐related biomarkers and annual change in gait speed (m·s^−1^) over the 15‐year follow‐up, excluding those with cerebrovascular disease at baseline (*n* = 74).
**Table S7:** Associations between quartile‐based AD‐related biomarkers and annual change in gait speed (m·s^−1^) over the 15‐year follow‐up, excluding those with imputed biomarkers (*n* = 6 for A*β*42 [A*β*42/40], *n* = 13 for *p*‐Tau181, *n* = 3 for *p*‐Tau217, *n* = 12 for *t‐*Tau).
**T** **able S8:** Associations between quartile‐based AD‐related biomarkers and annual change in gait speed (m·s^−1^) over the 15‐year follow‐up, excluding those with incident dementia (*n* = 266).

## Data Availability

The data originate from the SNAC‐K project, a population‐based study focused on aging and dementia (http://www.snac‐k.se/). Researchers can request access to these data upon approval from the SNAC‐K organization. Applications for access can be submitted via http://www.snac‐k.se/.
