# Supplementary material for: Blood Biomarkers of Alzheimer's Disease and Gait Speed Trajectories in Community‐Dwelling Older Adults: A Cohort Study
Source: J Cachexia Sarcopenia Muscle. 2026 Aug 18;17(4):e70365. doi: 10.1002/jcsm.70365 (PMC13487383; doi:10.1002/jcsm.70365)
Supplement: Supplementary file 1 [file JCSM-17-e70365-s001.docx]

**Supplementary table 1.** Eligible sample, excluded sample, and included sample.

|  | Eligible  sample  (n = 3,022) | | Excluded  sample  (n = 1,357) | | Included  sample  (n = 1,665) | | p-value |
| --- | --- | --- | --- | --- | --- | --- | --- |
| *Sociodemographic characteristics* |  |  |  |  |  |  |  |
| Age (years) , mean (SD) | 73.25 | (10.50) | 75.95 | (10.62) | 71.05 | (9.87) | **<0.0001** |
| BMI (kg·m^-2^), mean (SD) | 25.67 | (4.13) | 25.37 | (4.29) | 25.90 | (3.98) | **0.0007** |
| Female, n (%) | 1,905 | (63.04) | 864 | (63.67) | 1.041 | (62.52) | 0.54 |
| Education |  |  |  |  |  |  | **<0.0001** |
| Elementary, n (%) | 476 | (15.75) | 266 | (19.60) | 210 | (12.61) |  |
| High school, n (%) | 1496 | (49.50) | 689 | (50.77) | 807 | (48.47) |  |
| University, or above, n (%) | 1,042 | (34.48) | 394 | (29.03) | 648 | (38.92) |  |
| Civil status |  |  |  |  |  |  | **<0.0001** |
| Partnered, n (%) | 1,437 | (47.55) | 578 | (42.59) | 859 | (51.59) |  |
| Unpartnered, n (%) | 1,577 | (52.18) | 772 | (56.89) | 805 | (48.35) |  |
|  |  |  |  |  |  |  |  |
| *Physical activity level* |  |  |  |  |  |  | **<0.0001** |
| Inadequate, n (%) | 876 | (28.99) | 500 | (36.85) | 376 | (22.58) |  |
| Health or fitness-enhancing, n (%) | 2,146 | (71.01) | 857 | (63.15) | 1,289 | (77.42) |  |
|  |  |  |  |  |  |  |  |
| *Cognitive status* |  |  |  |  |  |  |  |
| MMSE score at baseline, mean (SD) | 28.57 | (2.04) | 28.14 | (2.50) | 28.92 | (1.48) | **<0.0001** |
|  |  |  |  |  |  |  |  |
| *Clinical assessments* |  |  |  |  |  |  |  |
| Number of chronic diseases, mean (SD) | 3.88 | (2.40) | 4.34 | (2.53) | 3.51 | (2.22) | **<0.0001** |
| Number of medications, mean (SD) | 3.77 | (3.29) | 4.27 | (3.44) | 3.37 | (3.11) | **<0.0001** |
| Depression and mood disorders, n (%) | 252 | (8.34) | 123 | (9.06) | 129 | (7.75) | 0.22 |
| Heart failure, n (%) | 267 | (8.84) | 172 | (12.68) | 95 | (5.71) | **<0.0001** |
| Ischemic heart disease, n (%) | 437 | (14.46) | 247 | (18.20) | 190 | (11.41) | **<0.0001** |
| Atrial fibrillation, n (%) | 268 | (8.87) | 165 | (12.16) | 103 | (6.19) | **<0.0001** |
| Other cardiovascular disease, n (%) | 93 | (3.08) | 62 | (4.57) | 31 | (1.86) | **<0.0001** |
| Hypertension, n (%) | 2,121 | (70.19) | 983 | (72.44) | 1,138 | (68.35) | **0.016** |
| Cerebrovascular disease, n (%) | 196 | (6.49) | 122 | (8.99) | 74 | (4.44) | **<0.0001** |
| Chronic kidney disease, n (%) | 998 | (33.02) | 519 | (38.25) | 479 | (28.77) | **<0.0001** |
| Diabetes, n (%) | 264 | (8.74) | 140 | (10.32) | 124 | (7.45) | **0.0067** |
| Obesity, n (%) | 385 | (12.74) | 155 | (11.42) | 230 | (13.81) | 0.057 |
| Asthma, COPD, emphysema, bronchitis, n (%) | 300 | (9.93) | 152 | (11.20) | 148 | (8.89) | **0.040** |
| Dorsopathies, n (%) | 201 | (6.65) | 88 | (6.48) | 113 | (6.79) | 0.80 |
| Inflammatory arthropathies, n (%) | 123 | (4.07) | 69 | (5.08) | 54 | (3.24) | **0.014** |
| Osteoarthritis degenerative joint disease, n (%) | 394 | (13.04) | 183 | (13.49) | 211 | (12.67) | 0.54 |
| Other musculoskeletal joint disease, n (%) | 182 | 6.02 | 92 | 6.78 | 90 | (5.41) | 0.13 |
| APOE ε4 carrier status, n (%) | 805 | (26.64) | 320 | (23.58) | 485 | (29.13) | **<0.0001** |
|  |  |  |  |  |  |  |  |
| *Physical function* |  |  |  |  |  |  |  |
| Gait speed at baseline (m·s^-1^), mean (SD) | 1.02 | (0.43) | 0.89 | (0.46) | 1.12 | (0.38) | **<0.0001** |
| ADLs |  |  |  |  |  |  | **<0.0001** |
| 0 difficulties, n (%) | 2,895 | (95.80) | 1,263 | (93.07) | 1,632 | (98.02) |  |
| ≥ 1 difficulties, n (%) | 119 | (3.94) | 86 | (6.34) | 33 | (1.98) |  |
|  |  |  |  |  |  |  |  |
| *AD biomarkers* |  |  |  |  |  |  |  |
| Aβ40 pg/mL, median [IQR] | 133.07 | [98.80-165.66] | 144.05 | [107.56-177.52] | 128.57 | [94.94-161.65] | **<0.0001** |
| Aβ42 pg/mL, median [IQR] | 7.30 | [5.53-9.07] | 7.65 | [5.93-9.34] | 7.18 | [5.44-8.96] | **<0.0001** |
| Aβ42/40, median [IQR] | 0.06 | [0.05-0.07] | 0.06 | [0.05-0.06] | 0.06 | [0.05-0.07] | **0.0027** |
| p-Tau181 pg/mL, median [IQR] | 1.17 | [0.74-1.78] | 1.43 | [0.84-2.24] | 1.09 | [0.72-1.66] | **<0.0001** |
| p-Tau217 pg/mL, median [IQR] | 0.10 | [0.06-0.18] | 0.13 | [0.07-0.25] | 0.09 | [0.05-0.16] | **<0.0001** |
| t-Tau pg/mL, median [IQR] | 0.84 | [0.54-1.18] | 0.94 | [0.60-1.36] | 0.81 | [0.53-1.13] | **<0.0001** |
| NfL pg/mL, median [IQR] | 17.82 | [12.50-27.99] | 22.82 | [14.56-36.81] | 16.70 | [12.09-25.29] | **<0.0001** |
| GFAP pg/mL, median [IQR] | 120.29 | [79.73-187.69] | 136.45 | [94.08-218.44] | 116.24 | [75.66-176.42] | **<0.0001** |

All variables are presented as Mean (SD) or n (%) or Median [IQR]. P values represent a comparison between excluded and included participants, with bold values indicating p < 0.05. All the statistical tests were two-sided. A mismatch between total numbers and sample sizes is due to missing data. Missing values for excluded participants: BMI = 97; civil status = 7; MMSE = 2; gait speed = 47; ADL = 8; APOE ε4 = 242; AD biomarkers = 782. Missing values for included participants: BMI = 30; civil status = 1; MMSE = 2; APOE ε4 = 11; AD biomarkers = 782. Aβ40 = amyloid-β42; Aβ42 = amyloid-β42; Aβ42/40 = amyloid-β42 to amyloid-β40 ratio; ADLs = activities of daily living; APOE ε4 = apolipoprotein E isoform 4; BMI = body mass index; GFAP = glial fibrillary acidic protein; MMSE = mini-mental state examination; NfL = neurofilament light chain; p-Tau181 = phosphorylated Tau181; p-Tau217 = phosphorylated Tau217; SD = standard deviation; t-Tau = total Tau.

**Supplementary table 2.** STROBE Statement—Checklist of items that should be included in reports of ***cohort studies***

|  | Item No | Recommendation | Page No |
| --- | --- | --- | --- |
| **Title and abstract** | 1 | (*a*) Indicate the study’s design with a commonly used term in the title or the abstract | 1 |
|  |  | (*b*) Provide in the abstract an informative and balanced summary of what was done and what was found | 3 |
| Introduction | | |  |
| Background/rationale | 2 | Explain the scientific background and rationale for the investigation being reported | 4 |
| Objectives | 3 | State specific objectives, including any prespecified hypotheses | 4 |
| Methods | | |  |
| Study design | 4 | Present key elements of study design early in the paper | 5 |
| Setting | 5 | Describe the setting, locations, and relevant dates, including periods of recruitment, exposure, follow-up, and data collection | 5 |
| Participants | 6 | (*a*) Give the eligibility criteria, and the sources and methods of selection of participants. Describe methods of follow-up | 5 |
|  |  | (*b*) For matched studies, give matching criteria and number of exposed and unexposed | - |
| Variables | 7 | Clearly define all outcomes, exposures, predictors, potential confounders, and effect modifiers. Give diagnostic criteria, if applicable | 6-8 |
| Data sources/ measurement | 8* | For each variable of interest, give sources of data and details of methods of assessment (measurement). Describe comparability of assessment methods if there is more than one group | 6-8 |
| Bias | 9 | Describe any efforts to address potential sources of bias | 8-9 |
| Study size | 10 | Explain how the study size was arrived at | 5 |
| Quantitative variables | 11 | Explain how quantitative variables were handled in the analyses. If applicable, describe which groupings were chosen and why | 8 |
| Statistical methods | 12 | (*a*) Describe all statistical methods, including those used to control for confounding | 8 |
|  |  | (*b*) Describe any methods used to examine subgroups and interactions | 8-9 |
|  |  | (*c*) Explain how missing data were addressed | 9 |
|  |  | (*d*) If applicable, explain how loss to follow-up was addressed |  |
|  |  | (*e*) Describe any sensitivity analyses | 9 |
| Results | | |  |
| Participants | 13* | (a) Report numbers of individuals at each stage of study—eg numbers potentially eligible, examined for eligibility, confirmed eligible, included in the study, completing follow-up, and analysed | 5, 9, Table 1, Suppl. Material |
|  |  | (b) Give reasons for non-participation at each stage | 5 |
|  |  | (c) Consider use of a flow diagram | Figure 1 |
| Descriptive data | 14* | (a) Give characteristics of study participants (eg demographic, clinical, social) and information on exposures and potential confounders | 9, Table 1, Suppl. Material |
|  |  | (b) Indicate number of participants with missing data for each variable of interest | Table 1, Suppl. Material |
|  |  | (c) Summarise follow-up time (eg, average and total amount) | 5, 9 |
| Outcome data | 15* | Report numbers of outcome events or summary measures over time | 5 |
| Main results | 16 | (*a*) Give unadjusted estimates and, if applicable, confounder-adjusted estimates and their precision (eg, 95% confidence interval). Make clear which confounders were adjusted for and why they were included | Without unadjusted estimates. Confunder-adjusted estimates: 9-10, Table 1, Suppl. Material |
|  |  | (*b*) Report category boundaries when continuous variables were categorized | 6, 8, Table 1, Suppl. Material |
|  |  | (*c*) If relevant, consider translating estimates of relative risk into absolute risk for a meaningful time period | - |
| Other analyses | 17 | Report other analyses done—eg analyses of subgroups and interactions, and sensitivity analyses | 10, Suppl. Material |
| Discussion | | |  |
| Key results | 18 | Summarise key results with reference to study objectives | 10-11 |
| Limitations | 19 | Discuss limitations of the study, taking into account sources of potential bias or imprecision. Discuss both direction and magnitude of any potential bias | 13 |
| Interpretation | 20 | Give a cautious overall interpretation of results considering objectives, limitations, multiplicity of analyses, results from similar studies, and other relevant evidence | 11-13 |
| Generalisability | 21 | Discuss the generalisability (external validity) of the study results | 13 |
| Other information | | |  |
| Funding | 22 | Give the source of funding and the role of the funders for the present study and, if applicable, for the original study on which the present article is based | 14 |

*Give information separately for exposed and unexposed groups.

**Note:** An Explanation and Elaboration article discusses each checklist item and gives methodological background and published examples of transparent reporting. The STROBE checklist is best used in conjunction with this article (freely available on the Web sites of PLoS Medicine at http://www.plosmedicine.org/, Annals of Internal Medicine at http://www.annals.org/, and Epidemiology at http://www.epidem.com/). Information on the STROBE Initiative is available at http://www.strobe-statement.org.

**Supplementary table 3.** Intra-assay and inter-assay precision expressed as the coefficient of variation (%).

|  | **Sample pool** | | | **Control 1** | | | **Control 2** | | |
| --- | --- | --- | --- | --- | --- | --- | --- | --- | --- |
| **Biomarker** | **Conc^1^** | **Within run^2^** | **Between run^3^** | **Conc^1^** | **Within run^2^** | **Between run^3^** | **Conc^1^** | **Within run^2^** | **Between run^3^** |
| Aβ42 | 6.6 | 4.8 | 7.4 | 3.4 | 6.1 | 8.2 | 76.8 | 2.1 | 4.7 |
| Aβ40 | 120.4 | 2.2 | 5.2 | 10.1 | 3.8 | 6.8 | 156.1 | 1.6 | 3.7 |
| t-Tau | 0.9 | 10.7 | 12.3 | 1.6 | 5.3 | 8.5 | 61.7 | 2.0 | 5.2 |
| p-Tau181 | 1.5 | 9.2 | 13.6 | 3.6 | 4.9 | 7.9 | 98.0 | 3.2 | 5.9 |
| p-Tau217 | 0.14 | 13.3 | 17.6 | 0.8 | 8.8 | 12.0 | 7.7 | 6.1 | 11.2 |
| NfL | 24.1 | 7.6 | 11.6 | 4.1 | 11.5 | 17.6 | 409.8 | 4.5 | 7.4 |
| GFAP | 194 | 13.1 | 21.4 | 89.8 | 11.4 | 17.4 | 10311 | 5.8 | 9.7 |

^1^Conc: average of the concentrations (pg/mL) obtained in 39 runs.

^2^Within run: coefficient of variation (%) calculated on the triplicate pool, control 1 and control 2 included in each run (plate). The average coefficient of variation for all runs is reported.

^3^Between run: coefficient of variation (%) calculated on the concentration values obtained for pool, control 1 and control 2 across all the runs (plate).

Aβ40 = amyloid-β40; Aβ42 = amyloid-β42; GFAP = glial fibrillary acidic protein; NfL = neurofilament light chain; p-Tau181 = phosphorylated Tau181; p-Tau217 = phosphorylated Tau217; t-Tau = total Tau.

**Supplementary table 4.** Associations between quartile-based AD-related biomarkers and annual change in gait speed (m·s^-1^) over the 15-year follow-up, with Bonferroni-adjusted p-values and CI for biomarker-by-follow-up time interaction terms.

|  | Model I | | | Model II | | | Model III | | |
| --- | --- | --- | --- | --- | --- | --- | --- | --- | --- |
|  | β | (98.3% CI) | p-value | β | (98.3% CI) | p-value | β | (98.3% CI) | p-value |
| Aβ42/40 |  |  |  |  |  |  |  |  |  |
| 1^st^ quartile | -0.002 | (-0.008; 0.004) | 1.00 | -0.002 | (-0.008; 0.004) | 1.00 | -0.001 | (-0.006; 0.005) | 1.00 |
| 2^nd^ quartile | 0.001 | (-0.004; 0.007) | 1.00 | 0.002 | (-0.004; 0.008) | 1.00 | 0.003 | (-0.003; 0.008) | 0.66 |
| 3^rd^ quartile | 0.003 | (-0.002; 0.009) | 0.40 | 0.003 | (-0.002; 0.009) | 0.41 | 0.003 | (-0.002; 0.009) | 0.33 |
| ^*^4^th^ quartile | - | - | - | - | - | - | - | - | - |
| p-Tau181 |  |  |  |  |  |  |  |  |  |
| ^*^1^st^ quartile | - | - | - | - | - | - | - | - | - |
| 2^nd^ quartile | 0.000 | (-0.006; 0.005) | 1.00 | 0.000 | (-0.006; 0.005) | 1.00 | 0.000 | (-0.005; 0.005) | 1.00 |
| 3^rd^ quartile | -0.005 | (-0.010; 0.001) | 0.10 | -0.004 | (-0.010; 0.001) | 0.16 | -0.004 | (-0.009; 0.002) | 0.29 |
| 4^th^ quartile | **-0.008** | **(-0.014; -0.002)** | **0.0083** | **-0.007** | **(-0.014; -0.001)** | **0.017** | -0.004 | (-0.010; 0.002) | 0.39 |
| p-Tau217 |  |  |  |  |  |  |  |  |  |
| ^*^1^st^ quartile | - | - | - | - | - | - | - | - | - |
| 2^nd^ quartile | -0.002 | (-0.008; 0.003) | 0.96 | -0.002 | (-0.008; 0.003) | 0.97 | -0.002 | (-0.007; 0.003) | 1.00 |
| 3^rd^ quartile | -0.002 | (-0.007; 0.004) | 1.00 | -0.001 | (-0.007; 0.004) | 1.00 | -0.001 | (-0.006; 0.004) | 1.00 |
| 4^th^ quartile | **-0.010** | **(-0.017; -0.004)** | **0.0006** | **-0.009** | **(-0.016; -0.003)** | **0.0023** | -0.005 | (-0.011; 0.001) | 0.16 |
| t-Tau |  |  |  |  |  |  |  |  |  |
| ^*^1^st^ quartile | - | - | - | - | - | - | - | - | - |
| 2^nd^ quartile | 0.000 | (-0.005; 0.006) | 1.00 | 0.000 | (-0.005; 0.006) | 1.00 | 0.000 | (-0.005; 0.005) | 1.00 |
| 3^rd^ quartile | -0.001 | (-0.007; 0.004) | 1.00 | -0.001 | (-0.007; 0.004) | 1.00 | -0.001 | (-0.007; 0.004) | 1.00 |
| 4^th^ quartile | 0.000 | (-0.006; 0.006) | 1.00 | 0.000 | (-0.006; 0.006) | 1.00 | 0.000 | (-0.005; 0.006) | 1.00 |
| NfL |  |  |  |  |  |  |  |  |  |
| ^*^1^st^ quartile | - | - | - | - | - | - | - | - | - |
| 2^nd^ quartile | -0.003 | (-0.009; 0.002) | 0.42 | -0.003 | (-0.008; 0.002) | 0.49 | -0.003 | (-0.008; 0.002) | 0.36 |
| 3^th^ quartile | **-0.008** | **(-0.014; -0.002)** | **0.0039** | **-0.008** | **(-0.014; -0.002)** | **0.0041** | **-0.007** | **(-0.013; -0.001)** | **0.0090** |
| 4^th^ quartile | **-0.013** | **(-0.021; -0.005)** | **0.0004** | **-0.012** | **(-0.020; -0.004)** | **0.0007** | **-0.008** | **(-0.015; 0.000)** | **0.043** |
| GFAP |  |  |  |  |  |  |  |  |  |
| ^*^1^st^ quartile | - | - | - | - | - | - | - | - | - |
| 2^nd^ quartile | 0.001 | (-0.004; 0.006) | 1.00 | 0.001 | (-0.008; 0.002) | 1.00 | 0.001 | (-0.004; 0.006) | 1.00 |
| 3^th^ quartile | -0.002 | (-0.008; 0.004) | 1.00 | -0.002 | (-0.014; -0.002) | 1.00 | -0.001 | (-0.007; 0.004) | 1.00 |
| 4^th^ quartile | **-0.007** | **(-0.014; 0.000)** | **0.032** | **-0.007** | **(-0.020; -0.004)** | **0.040** | -0.003 | (-0.010; 0.003) | 0.70 |

This table depicts Bonferroni-adjusted results from linear mixed-effect models with longitudinal gait speed (m·s^-1^) as the outcome and quartile-based AD-related biomarkers as predictors.

Aβ42/40 = amyloid-β42 to amyloid-β40 ratio; CI = confidence interval; GFAP = glial fibrillary acidic protein; NfL = neurofilament light; p-Tau181 = phosphorylated Tau181; p-Tau217 = phosphorylated Tau217; t-tau = total Tau.

Model I: model including age, sex, education, BMI, physical activity, number of drugs, chronic kidney disease, diabetes, hypertension, cerebrovascular disease, heart failure, atrial fibrillation, ischemic heart disease, and other cardiovascular diseases.

Model II: model I + APOE ε4.

Model III: model II + time-varying Mini-Mental State Examination scale score.

Bold indicates p < 0.05.

*Reference quartile.

**Supplementary table 5.** Associations between quartile-based AD-related biomarkers and annual change in gait speed (m·s^-1^) over the 15-year follow-up, excluding those participants with GS < 0.8 m·s^-1^ at baseline (n = 268).

|  | Model I | | | Model II | | | Model III | | |
| --- | --- | --- | --- | --- | --- | --- | --- | --- | --- |
|  | β | (95% CI) | p-value | β | (95% CI) | p-value | β | (95% CI) | p-value |
| Aβ42/40 |  |  |  |  |  |  |  |  |  |
| 1^st^ quartile | -0.002 | (-0.007; 0.003) | 0.43 | -0.002 | (-0.006; 0.003) | 0.51 | -0.001 | (-0.005; 0.004) | 0.80 |
| 2^nd^ quartile | 0.001 | (-0.004; 0.005) | 0.83 | 0.001 | (-0.004; 0.006) | 0.69 | 0.002 | (-0.003; 0.006) | 0.45 |
| 3^rd^ quartile | **0.005** | **(0.000; 0.009)** | **0.045** | **0.005** | **(0.000; 0.009)** | **0.044** | 0.004 | (0.000; 0.009) | 0.057 |
| ^*^4^th^ quartile | - | - | - | - | - | - | - | - | - |
| p-Tau181 |  |  |  |  |  |  |  |  |  |
| ^*^1^st^ quartile | - | - | - | - | - | - | - | - | - |
| 2^nd^ quartile | 0.002 | (-0.003; 0.006) | 0.53 | 0.002 | (-0.003; 0.006) | 0.49 | 0.002 | (-0.003; 0.006) | 0.44 |
| 3^rd^ quartile | -0.004 | (-0.009; 0.000) | 0.070 | -0.004 | (-0.009; 0.001) | 0.088 | -0.003 | (-0.007; 0.001) | 0.19 |
| 4^th^ quartile | **-0.008** | **(-0.013; -0.002)** | **0.0041** | **-0.007** | **(-0.012; -0.002)** | **0.0076** | -0.004 | (-0.009; 0.001) | 0.12 |
| p-Tau217 |  |  |  |  |  |  |  |  |  |
| ^*^1^st^ quartile | - | - | - | - | - | - | - | - | - |
| 2^nd^ quartile | 0.000 | (-0.004; 0.005) | 0.86 | 0.000 | (-0.004; 0.005) | 0.84 | 0.001 | (-0.004; 0.005) | 0.75 |
| 3^rd^ quartile | -0.001 | (-0.005; 0.004) | 0.82 | 0.000 | (-0.005; 0.004) | 0.85 | 0.000 | (-0.005; 0.004) | 0.96 |
| 4^th^ quartile | **-0.012** | **(-0.017; -0.006)** | **<0.0001** | **-0.011** | **(-0.016; -0.006)** | **<0.0001** | **-0.007** | **(-0.012; -0.002)** | **0.0070** |
| t-Tau |  |  |  |  |  |  |  |  |  |
| ^*^1^st^ quartile | - | - | - | - | - | - | - | - | - |
| 2^nd^ quartile | 0.002 | (-0.002; 0.007) | 0.35 | 0.002 | (-0.002; 0.007) | 0.33 | 0.002 | (-0.002; 0.007) | 0.32 |
| 3^rd^ quartile | -0.002 | (-0.007; 0.003) | 0.42 | -0.002 | (-0.007; 0.003) | 0.40 | -0.001 | (-0.006; 0.003) | 0.54 |
| 4^th^ quartile | 0.000 | (-0.005; 0.004) | 0.91 | 0.000 | (-0.005; 0.004) | 0.88 | 0.000 | (-0.005; 0.004) | 0.93 |
| NfL |  |  |  |  |  |  |  |  |  |
| ^*^1^st^ quartile | - | - | - | - | - | - | - | - | - |
| 2^nd^ quartile | -0.002 | (-0.007; 0.002) | 0.34 | -0.002 | (-0.007; 0.003) | 0.39 | -0.002 | (-0.007; 0.002) | 0.34 |
| 3^th^ quartile | **-0.007** | **(-0.012; -0.002)** | **0.0074** | **-0.007** | **(-0.012; -0.002)** | **0.0086** | **-0.006** | **(-0.011; -0.001)** | **0.011** |
| 4^th^ quartile | **-0.014** | **(-0.020; -0.007)** | **<0.0001** | **-0.013** | **(-0.019; -0.007)** | **<0.0001** | **-0.009** | **(-0.015; -0.003)** | **0.0031** |
| GFAP |  |  |  |  |  |  |  |  |  |
| ^*^1^st^ quartile | - | - | - | - | - | - | - | - | - |
| 2^nd^ quartile | 0.001 | (-0.003; 0.006) | 0.54 | 0.001 | (-0.003; 0.006) | 0.59 | 0.001 | (-0.003; 0.006) | 0.56 |
| 3^th^ quartile | 0.001 | (-0.004; 0.005) | 0.81 | 0.001 | (-0.004; 0.005) | 0.78 | 0.001 | (-0.004; 0.005) | 0.77 |
| 4^th^ quartile | **-0.008** | **(-0.014; -0.003)** | **0.0047** | **-0.008** | **(-0.013; -0.002)** | **0.0065** | -0.004 | (-0.010; 0.001) | 0.11 |

This table depicts the results from linear mixed-effect models with longitudinal gait speed (m·s^-1^) as the outcome and quartile-based AD-related biomarkers as predictors in fast gait speed participants.

Aβ42/40 = amyloid-β42 to amyloid-β40 ratio; CI = confidence interval; GFAP = glial fibrillary acidic protein; NfL = neurofilament light; p-Tau181 = phosphorylated Tau181; p-Tau217 = phosphorylated Tau217; t-tau = total Tau.

Model I: model including age, sex, education, BMI, physical activity, number of drugs, chronic kidney disease, diabetes, hypertension, cerebrovascular disease, heart failure, atrial fibrillation, ischemic heart disease, and other cardiovascular diseases.

Model II: model I + APOE ε4.

Model III: model II + time-varying Mini-Mental State Examination scale score.

Bold indicates p < 0.05.

*Reference quartile.

**Supplementary table 6.** Associations between quartile-based AD-related biomarkers and annual change in gait speed (m·s^-1^) over the 15-year follow-up, excluding those with cerebrovascular disease at baseline (n = 74).

|  | Model I | | | Model II | | | Model III | | |
| --- | --- | --- | --- | --- | --- | --- | --- | --- | --- |
|  | β per year | (95% CI) | p-value | β per year | (95% CI) | p-value | β per year | (95% CI) | p-value |
| Aβ42/40 |  |  |  |  |  |  |  |  |  |
| 1^st^ quartile | -0.003 | (-0.007; 0.002) | 0.27 | -0.002 | (-0.007; 0.002) | 0.32 | -0.002 | (-0.006; 0.003) | 0.51 |
| 2^nd^ quartile | 0.001 | (-0.004; 0.005) | 0.78 | 0.001 | (-0.003; 0.006) | 0.62 | 0.002 | (-0.002; 0.006) | 0.38 |
| 3^rd^ quartile | 0.003 | (-0.002; 0.007) | 0.21 | 0.003 | (-0.002; 0.007) | 0.22 | 0.003 | (-0.001; 0.007) | 0.18 |
| ^*^4^th^ quartile | - | - | - | - | - | - | - | - | - |
| p-Tau181 |  |  |  |  |  |  |  |  |  |
| ^*^1^st^ quartile | - | - | - | - | - | - | - | - | - |
| 2^nd^ quartile | -0.001 | (-0.005; 0.003) | 0.65 | -0.001 | (-0.005; 0.003) | 0.68 | -0.001 | (-0.005; 0.004) | 0.78 |
| 3^rd^ quartile | **-0.005** | **(-0.009; -0.001)** | **0.045** | -0.004 | (-0.009; 0.001) | 0.063 | -0.004 | (-0.008; 0.001) | 0.10 |
| 4^th^ quartile | **-0.009** | **(-0.014; -0.003)** | **0.0014** | **-0.008** | **(-0.013; -0.003)** | **0.0030** | -0.004 | (-0.009; 0.001) | 0.10 |
| p-Tau217 |  |  |  |  |  |  |  |  |  |
| ^*^1^st^ quartile | - | - | - | - | - | - | - | - | - |
| 2^nd^ quartile | -0.002 | (-0.007; 0.002) | 0.32 | -0.002 | (-0.007; 0.002) | 0.32 | -0.002 | (-0.006; 0.003) | 0.42 |
| 3^rd^ quartile | -0.003 | (-0.007; 0.002) | 0.28 | -0.002 | (-0.007; 0.002) | 0.34 | -0.002 | (-0.006; 0.003) | 0.48 |
| 4^th^ quartile | **-0.010** | **(-0.015; -0.004)** | **0.0004** | **-0.009** | **(-0.014; -0.004)** | **0.0013** | -0.005 | (-0.010; 0.001) | 0.079 |
| t-Tau |  |  |  |  |  |  |  |  |  |
| ^*^1^st^ quartile | - | - | - | - | - | - | - | - | - |
| 2^nd^ quartile | 0.000 | (-0.005; 0.004) | 0.97 | 0.000 | (-0.005; 0.005) | 1.00 | 0.000 | (-0.005; 0.004) | 0.91 |
| 3^rd^ quartile | -0.002 | (-0.007; 0.002) | 0.35 | -0.002 | (-0.007; 0.002) | 0.34 | -0.002 | (-0.006; 0.002) | 0.39 |
| 4^th^ quartile | -0.001 | (-0.005; 0.004) | 0.81 | -0.001 | (-0.005; 0.004) | 0.79 | 0.000 | (-0.005; 0.004) | 0.83 |
| NfL |  |  |  |  |  |  |  |  |  |
| ^*^1^st^ quartile | - | - | - | - | - | - | - | - | - |
| 2^nd^ quartile | -0.003 | (-0.007; 0.001) | 0.16 | -0.003 | (-0.007; 0.001) | 0.20 | -0.003 | (-0.007; 0.001) | 0.16 |
| 3^th^ quartile | **-0.008** | **(-0.013; -0.003)** | **0.0017** | **-0.008** | **(-0.013; -0.003)** | **0.0018** | **-0.007** | **(-0.011; -0.002)** | **0.0057** |
| 4^th^ quartile | **-0.013** | **(-0.020; -0.006)** | **0.0001** | **-0.012** | **(-0.019; -0.006)** | **0.0002** | **-0.008** | **(-0.015; -0.002)** | **0.0011** |
| GFAP |  |  |  |  |  |  |  |  |  |
| ^*^1^st^ quartile | - | - | - | - | - | - | - | - | - |
| 2^nd^ quartile | 0.001 | (-0.004; 0.005) | 0.78 | 0.001 | (-0.004; 0.005) | 0.80 | 0.001 | (-0.004; 0.005) | 0.82 |
| 3^th^ quartile | -0.003 | (-0.007; 0.002) | 0.27 | -0.003 | (-0.007; 0.002) | 0.28 | -0.002 | (-0.006; 0.003) | 0.47 |
| 4^th^ quartile | **-0.008** | **(-0.014; -0.002)** | **0.0059** | **-0.008** | **(-0.013; -0.002)** | **0.0080** | -0.004 | (-0.009; 0.002) | 0.16 |

This table depicts the results from linear mixed-effect models with longitudinal gait speed (m·s^-1^) as the outcome and quartile-based AD-related biomarkers as predictors in participants without cerebrovascular disease.

Aβ42/40 = amyloid-β42 to amyloid-β40 ratio; CI = confidence interval; GFAP = glial fibrillary acidic protein; NfL = neurofilament light chain; p-Tau181 = phosphorylated Tau181; p-Tau217 = phosphorylated Tau217; t-tau = total Tau.

Model I: model including age, sex, education, BMI, physical activity, number of drugs, chronic kidney disease, diabetes, hypertension, cerebrovascular disease, heart failure, atrial fibrillation, ischemic heart disease, and other cardiovascular diseases.

Model II: model I + APOE ε4.

Model III: model II + time-varying Mini-Mental State Examination scale score.

Bold indicates p < 0.05.

*Reference quartile.

**Supplementary table 7.** Associations between quartile-based AD-related biomarkers and annual change in gait speed (m·s^-1^) over the 15-year follow-up, excluding those with imputed biomarkers (n = 6 for Aβ42 [Aβ42/40], n = 13 for p-Tau181, n = 3 for p-Tau217, n = 12 for t-Tau).

|  | Model I | | | Model II | | | Model III | | |
| --- | --- | --- | --- | --- | --- | --- | --- | --- | --- |
|  | β | (95% CI) | p-value | β | (95% CI) | p-value | β | (95% CI) | p-value |
| Aβ42/40 |  |  |  |  |  |  |  |  |  |
| 1^st^ quartile | -0.002 | (-0.007; 0.002) | 0.32 | -0.002 | (-0.007; 0.003) | 0.38 | -0.001 | (-0.006; 0.003) | 0.655 |
| 2^nd^ quartile | 0.001 | (-0.003; 0.006) | 0.56 | 0.002 | (-0.003; 0.006) | 0.43 | 0.003 | (-0.002; 0.007) | 0.224 |
| 3^rd^ quartile | 0.004 | (-0.001; 0.008) | 0.11 | 0.004 | (-0.001; 0.008) | 0.12 | 0.004 | (-0.001; 0.008) | 0.091 |
| ^*^4^th^ quartile | - | - | - | - | - | - | - | - | - |
| p-Tau181 |  |  |  |  |  |  |  |  |  |
| ^*^1^st^ quartile | - | - | - | - | - | - | - | - | - |
| 2^nd^ quartile | 0.000 | (-0.005; 0.004) | 0.86 | 0.000 | (-0.005; 0.004) | 0.90 | 0.000 | (-0.004; 0.004) | 0.97 |
| 3^rd^ quartile | **-0.005** | **(-0.009; 0.000)** | **0.040** | -0.004 | (-0.009; 0.000) | 0.060 | -0.003 | (-0.008; 0.001) | 0.12 |
| 4^th^ quartile | **-0.008** | **(-0.013; -0.003)** | **0.0027** | **-0.008** | **(-0.013; -0.002)** | **0.0052** | -0.004 | (-0.009; 0.001) | 0.13 |
| p-Tau217 |  |  |  |  |  |  |  |  |  |
| ^*^1^st^ quartile | - | - | - | - | - | - | - | - | - |
| 2^nd^ quartile | -0.002 | (-0.007; 0.002) | 0.33 | -0.002 | (-0.007; 0.002) | 0.33 | -0.002 | (-0.006; 0.003) | 0.45 |
| 3^rd^ quartile | -0.001 | (-0.006; 0.003) | 0.51 | -0.001 | (-0.006; 0.003) | 0.60 | -0.001 | (-0.005; 0.004) | 0.76 |
| 4^th^ quartile | **-0.010** | **(-0.015; -0.005)** | **0.0003** | **-0.009** | **(-0.015; -0.004)** | **0.0009** | -0.005 | (-0.010; 0.000) | 0.062 |
| t-Tau |  |  |  |  |  |  |  |  |  |
| ^*^1^st^ quartile | - | - | - | - | - | - | - | - | - |
| 2^nd^ quartile | 0.000 | (-0.004; 0.005) | 0.85 | 0.001 | (-0.004; 0.005) | 0.82 | 0.000 | (-0.004; 0.004) | 0.97 |
| 3^rd^ quartile | -0.002 | (-0.006; 0.003) | 0.47 | -0.002 | (-0.006; 0.003) | 0.45 | -0.002 | (-0.006; 0.003) | 0.47 |
| 4^th^ quartile | 0.000 | (-0.005; 0.005) | 0.98 | 0.000 | (-0.005; 0.005) | 0.96 | 0.000 | (-0.004; 0.004) | 0.99 |

This table depicts the results from linear mixed-effect models with longitudinal gait speed (m·s^-1^) as the outcome and quartile-based AD-related biomarkers as predictors in participants without imputed biomarkers.

Aβ42/40 = amyloid-β42 to amyloid-β40 ratio; CI = confidence interval; p-Tau181 = phosphorylated Tau181; p-Tau217 = phosphorylated Tau217; t-tau = total Tau.

Model I: model including age. sex. education. BMI. physical activity. number of drugs. chronic kidney disease. diabetes. hypertension. cerebrovascular disease. heart failure. atrial fibrillation. ischemic heart disease. and other cardiovascular diseases.

Model II: model I + APOE ε4.

Model III: model II + time-varying Mini-Mental State Examination scale score.

Bold indicates p < 0.05.

*Reference quartile.

**Supplementary table 8.** Associations between quartile-based AD-related biomarkers and annual change in gait speed (m·s^-1^) over the 15-year follow-up, excluding those with incident dementia (n = 266).

|  | Model I | | | Model II | | | Model III | | |
| --- | --- | --- | --- | --- | --- | --- | --- | --- | --- |
|  | β | (95% CI) | p-value | β | (95% CI) | p-value | β | (95% CI) | p-value |
| Aβ42/40 |  |  |  |  |  |  |  |  |  |
| 1^st^ quartile | -0.001 | (-0.005; 0.004) | 0.82 | -0.001 | (-0.006; 0.004) | 0.74 | 0.000 | (-0.005; 0.004) | 0.88 |
| 2^nd^ quartile | 0.002 | (-0.003; 0.006) | 0.47 | 0.002 | (-0.003; 0.006) | 0.46 | 0.002 | (-0.002; 0.007) | 0.35 |
| 3^rd^ quartile | **0.005** | **(0.001; 0.009)** | **0.030** | **0.005** | **(0.000; 0.009)** | **0.031** | **0.005** | **(0.001; 0.009)** | **0.025** |
| ^*^4^th^ quartile | - | - | - | - | - | - | - | - | - |
| p-Tau181 |  |  |  |  |  |  |  |  |  |
| ^*^1^st^ quartile |  |  |  |  |  |  |  |  |  |
| 2^nd^ quartile | 0.000 | (-0.005; 0.004) | 0.84 | 0.000 | (-0.005; 0.004) | 0.84 | 0.000 | (-0.005; 0.004) | 0.86 |
| 3^rd^ quartile | -0.004 | (-0.009; 0.000) | 0.077 | -0.004 | (-0.008; 0.000) | 0.084 | -0.003 | (-0.008; 0.001) | 0.14 |
| 4^th^ quartile | -0.005 | (-0.010; 0.000) | 0.066 | -0.005 | (-0.010; 0.001) | 0.080 | -0.004 | (-0.009; 0.001) | 0.13 |
| p-Tau217 |  |  |  |  |  |  |  |  |  |
| ^*^1^st^ quartile | - | - | - | - | - | - | - | - | - |
| 2^nd^ quartile | 0.000 | (-0.004; 0.005) | 0.97 | 0.000 | (-0.005; 0.004) | 0.98 | 0.000 | (-0.004; 0.005) | 0.97 |
| 3^rd^ quartile | -0.001 | (-0.006; 0.003) | 0.55 | -0.002 | (-0.006; 0.003) | 0.51 | -0.001 | (-0.005; 0.004) | 0.68 |
| 4^th^ quartile | -0.005 | (-0.010; 0.000) | 0.061 | -0.005 | (-0.010; 0.000) | 0.072 | -0.004 | (-0.009; 0.001) | 0.13 |
| t-Tau |  |  |  |  |  |  |  |  |  |
| ^*^1^st^ quartile | - | - | - | - | - | - | - | - | - |
| 2^nd^ quartile | -0.001 | (-0.005; 0.004) | 0.75 | -0.001 | (-0.006; 0.004) | 0.69 | -0.001 | (-0.005; 0.004) | 0.74 |
| 3^rd^ quartile | -0.003 | (-0.008; 0.002) | 0.23 | -0.003 | (-0.008; 0.002) | 0.22 | -0.003 | (-0.007; 0.002) | 0.28 |
| 4^th^ quartile | 0.000 | (-0.005; 0.004) | 0.85 | -0.001 | (-0.005; 0.004) | 0.81 | -0.001 | (-0.005; 0.004) | 0.79 |
| NfL |  |  |  |  |  |  |  |  |  |
| ^*^1^st^ quartile | - | - | - | - | - | - | - | - | - |
| 2^nd^ quartile | -0.002 | (-0.007; 0.002) | 0.31 | -0.002 | (-0.007; 0.002) | 0.32 | -0.002 | (-0.007; 0.002) | 0.34 |
| 3^th^ quartile | **-0.008** | **(-0.013; -0.003)** | **0.0010** | **-0.008** | **(-0.013; -0.003)** | **0.0009** | **-0.007** | **(-0.012 -0.003)** | **0.0024** |
| 4^th^ quartile | **-0.007** | **(-0.014; -0.001)** | **0.021** | **-0.007** | **(-0.014; -0.001)** | **0.025** | -0.006 | (-0.012. 0.001) | 0.083 |
| GFAP |  |  |  |  |  |  |  |  |  |
| ^*^1^st^ quartile | - | - | - | - | - | - | - | - | - |
| 2^nd^ quartile | 0.001 | (-0.004; 0.005) | 0.78 | 0.001 | (-0.004; 0.005) | 0.81 | 0.001 | (-0.004; 0.005) | 0.71 |
| 3^th^ quartile | 0.000 | (-0.005; 0.004) | 0.88 | 0.000 | (-0.005; 0.004) | 0.92 | 0.000 | (-0.005; 0.005) | 0.98 |
| 4^th^ quartile | -0.003 | (-0.009; 0.003) | 0.30 | -0.003 | (-0.008; 0.003) | 0.33 | -0.001 | (-0.007; 0.004) | 0.64 |

This table depicts the results from linear mixed-effect models with longitudinal gait speed (m·s^-1^) as the outcome and quartile-based AD-related biomarkers as predictors in participants without incident dementia.

Aβ42/40 = amyloid-β42 to amyloid-β40 ratio; CI = confidence interval; GFAP = glial fibrillary acidic protein; NfL = neurofilament light chain; p-Tau181 = phosphorylated Tau181; p-Tau217 = phosphorylated Tau217; t-tau = total Tau.

Model I: model including age. sex. education. BMI. physical activity. number of drugs. chronic kidney disease. diabetes. hypertension. cerebrovascular disease. heart failure. atrial fibrillation. ischemic heart disease. and other cardiovascular diseases.

Model II: model I + APOE ε4.

Model III: model II + time-varying Mini-Mental State Examination scale score.

Bold indicates p < 0.05.

*Reference quartile.
